# Focal Nodular Hyperplasia Presenting With Biliary Obstruction and Intrahepatic Gallstones: A Case Report and Literature Review

**DOI:** 10.7759/cureus.110066

**Published:** 2026-06-01

**Authors:** Tunde Oyebamiji, Robin Schmitz, Anthony Gebran, Bassem S Hendawy, Samer T Tohme

**Affiliations:** 1 Department of Surgery, University of Pittsburgh Medical Center, Pittsburgh, USA; 2 Department of Pathology, University of Pittsburgh Medical Center, Pittsburgh, USA

**Keywords:** benign liver tumor, biliary obstruction, focal nodular hyperplasia (fnh), hepatolithiasis, intrahepatic gallstones

## Abstract

Focal nodular hyperplasia (FNH) is a benign liver lesion that is most often asymptomatic and does not require surgical intervention. Rarely, FNH may cause biliary obstruction and stasis, which can lead to intrahepatic gallstone (hepatolithiasis) formation. We describe a case of a 44-year-old female patient who was incidentally found to have biliary ductal dilation with an associated malignant-appearing 3.5 cm left liver mass. Workup included contrast-enhanced computed tomography (CT) and magnetic resonance imaging (MRI). A biopsy was deferred, given the high clinical suspicion. An open anatomic left hepatectomy was performed without complications, and the patient recovered well. Pathology demonstrated a 3.5 cm FNH with extensive intrahepatic cholelithiasis but no malignancy. Despite high-quality preoperative imaging, FNH could not be distinguished from intrahepatic cholangiocarcinoma, underscoring the diagnostic challenge posed by atypically presenting benign hepatic lesions. Surgical resection was indicated, given the biliary obstruction, stone burden, and radiologic suspicion for malignancy, and was performed safely with favorable outcomes.

## Introduction

Biliary obstruction and intrahepatic gallstones (hepatolithiasis) are well-recognized hepatobiliary conditions characterized by cholestasis manifesting as jaundice and pruritus, right upper quadrant pain, and sometimes recurrent episodes of cholangitis [1]. Most cases are attributed to intrinsic lesions such as choledocholithiasis, benign or malignant biliary strictures, and cholangiocarcinoma, resulting in chronic bile stasis [2-3]. While intrahepatic gallstones are more commonly reported in East and Southeast Asia, their occurrence remains rare in Western populations [2]. Although bile duct obstruction is typically caused by intraluminal or mural pathologies, it may occasionally arise from extrinsic compression by adjacent benign or malignant masses. Reported examples include pancreatic pseudocysts, duodenal diverticula, and lymphadenopathy [4-6]. Compression of the bile ducts by benign hepatic tumors is very rare.

Focal nodular hyperplasia (FNH) is a benign liver lesion that accounts for 0.6%-3% of hepatic lesions detected on imaging [7-9]. It predominantly affects women of reproductive age and is often discovered incidentally during abdominal imaging [8,10]. Most cases are asymptomatic and do not require intervention [11]. However, in rare circumstances, particularly when lesions are large or located near the hepatic hilum, FNH can exert mass effect on adjacent vascular or biliary structures, resulting in clinical symptoms [7].

A comprehensive literature search using PubMed, Medline, and Google Scholar identified only one published case report of FNH causing compression of the biliary system [12], specifically at the level of the cystic duct, but no reports documenting intrahepatic biliary obstruction or intrahepatic stone formation in this context. Here, we report a rare case of a small FNH presenting with biliary obstruction and intrahepatic stone formation, highlighting a unique complication of a typically indolent benign hepatic tumor.

## Case presentation

A 44-year-old Caucasian woman with a medical history notable for hypertension, hyperlipidemia, gastroesophageal reflux disease, and obesity (BMI 35.6 kg/m^2^) presented to our clinic with incidentally found biliary ductal dilation of the left lobe and a 3.2 cm liver mass concerning for malignancy. She originally underwent computed tomography (CT) of the chest to screen for coronary artery disease based on her comorbidities and positive family history. On this CT, she was incidentally found to have left hepatic lobe biliary ductal dilation. Her liver function tests were within normal limits, including a total bilirubin level of 0.6 mg/dL, aspartate aminotransferase (AST) 11 U/L, alanine aminotransferase (ALT) 26 U/L, alkaline phosphatase (ALP) 68 U/L, and gamma-glutamyl transferase (GGT) 60 U/L. Follow-up magnetic resonance imaging (MRI) of the abdomen with and without contrast re-demonstrated a 3.2 cm liver lesion centered in the left lobe at the hepatic hilum, abutting the left intrahepatic bile duct, with biliary ductal dilation concerning for malignancy based on the signal intensities on T2- and T1-weighted images, as well as the enhancement pattern with peripheral rim enhancement on the arterial phase and fill-in on delayed images. Additionally, a 4.1 cm right hepatic lobe hemangioma was identified (Figure 1).

**Figure 1:**
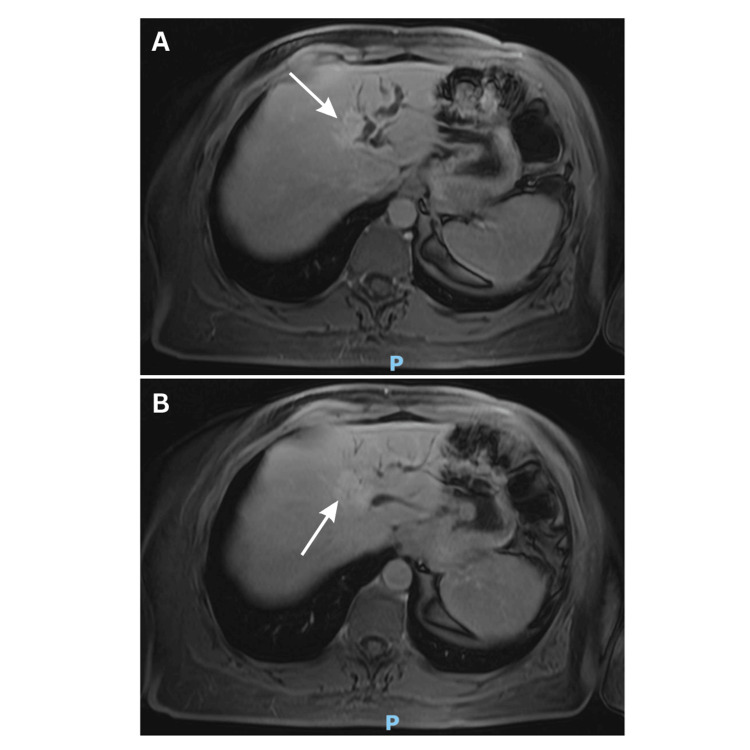
Preoperative liver magnetic resonance imaging with and without contrast, with the axial post-contrast image demonstrating a 3.2 cm central liver lesion with associated left-lobe biliary ductal dilation, highlighted by the white arrow (A). Axial post-contrast image at a slightly different level demonstrating the same lesion, highlighted by the white arrow (B).

Notably, no intrahepatic stones were identified on CT, MRI, or magnetic resonance cholangiopancreatography (MRCP) sequences performed as part of the same examination. Tumor markers were collected, including CEA and CA19-9, which were both normal. She reported intentional weight loss over the preceding two years. She denied abdominal pain or GI symptoms except for acid reflux that was treated with a proton pump inhibitor. She had no history of prior surgery and a negative family history for GI cancers. Colonoscopy and upper endoscopy were performed approximately eight months before presentation and did not show any upper or lower GI cancers.

We discussed with the patient the risks and benefits of percutaneous biopsy; however, given the imaging features highly suspicious for intrahepatic cholangiocarcinoma, including peripheral rim enhancement, delayed fill-in, and associated segmental biliary ductal dilation, and the presence of a resectable lesion, we elected to proceed directly to open surgical resection without prior tissue sampling. Intraoperative ultrasound demonstrated an ill-defined mass as well as intrahepatic gallstones, and we elected to pursue an anatomic left hepatic lobectomy. A cholecystectomy was also performed due to the presence of gallstones and to facilitate a completion cholangiogram (Figure 2).

**Figure 2:**
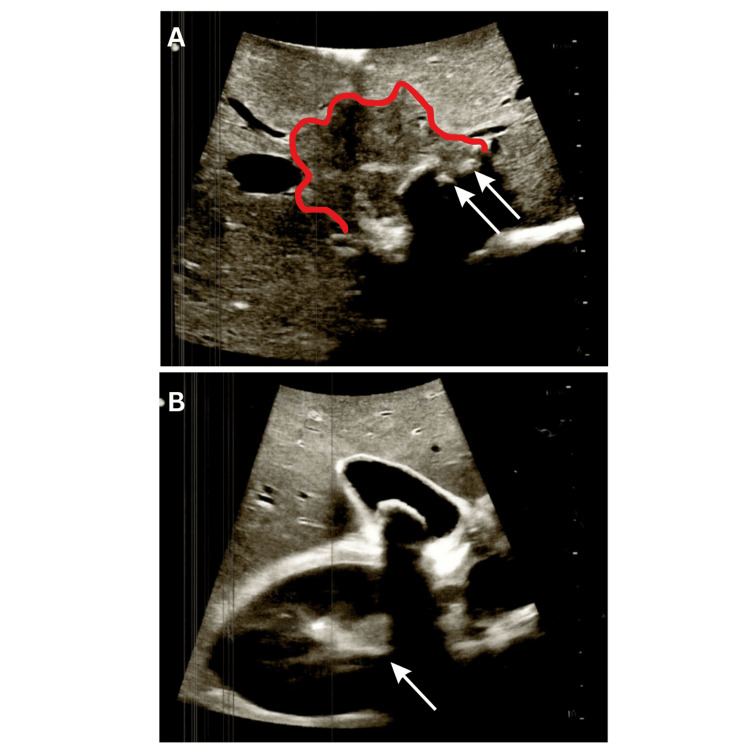
Intraoperative ultrasound showing an ill-defined hypoechoic liver lesion abutting the middle hepatic vein (red contour) causing biliary ductal dilation with intrahepatic gallstones (white arrows) (A). Gallbladder with a large gallstone (white arrow) (B).

The procedure was performed without any intraoperative complications. Once the specimen was removed, we bisected the specimen ex situ and confirmed the presence of a small hepatic mass. A large burden of intrahepatic gallstones was seen that were not identified on preoperative imaging (Figure 3).

**Figure 3:**
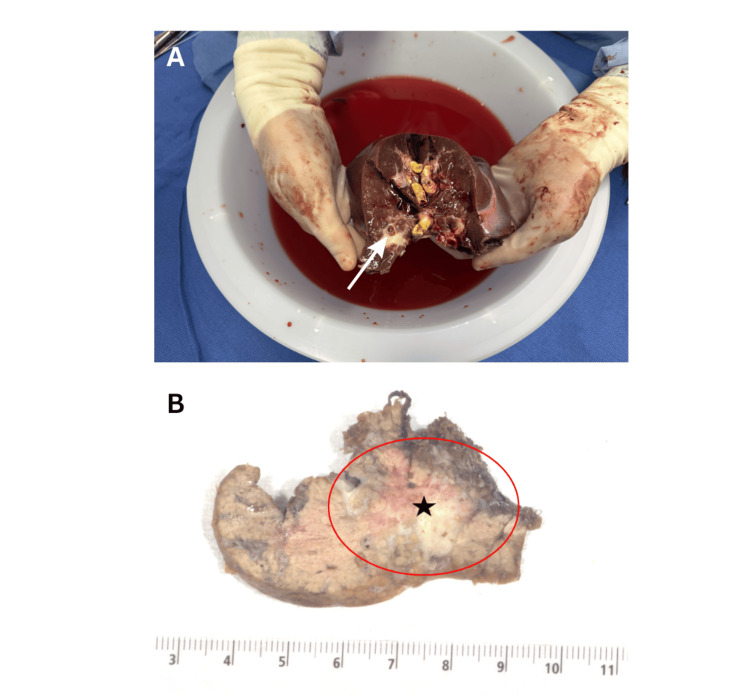
Gross intraoperative (A) and pathologic (B) specimens of the resected hepatic lesion. The lesion is well-circumscribed and demonstrates a central stellate scar (arrow in A; asterisk in B).

Histologic evaluation identified a 3.5 cm focal nodular hyperplasia with negative margins. The surrounding hepatic parenchyma showed characteristic nodular regenerative hyperplasia. The bile ducts had choledocholithiasis as demonstrated in the macroscopic pictures with surrounding chronic inflammation. There was no evidence of malignancy or dysplasia. Given this significant discrepancy between the clinical/radiology findings and the pathologic picture, the case was carefully reviewed in a multidisciplinary setting with an experienced GI radiologist, pathologist, and our surgical team. While the radiologic impression was highly suggestive of a malignant neoplasm (i.e., intrahepatic cholangiocarcinoma), there was no histologic evidence in the specimen to support such a suggestion. The findings were typical of FNH, including the central stellate scar with abnormal vessels surrounded by prominent nodular regenerative hyperplastic changes and a characteristic "map-like" appearance on glutamine synthetase immunohistochemical (IHC) stain that resulted in chronic segmental duct compression, bile stasis, and secondary intrahepatic stone formation (Figure 4).

**Figure 4:**
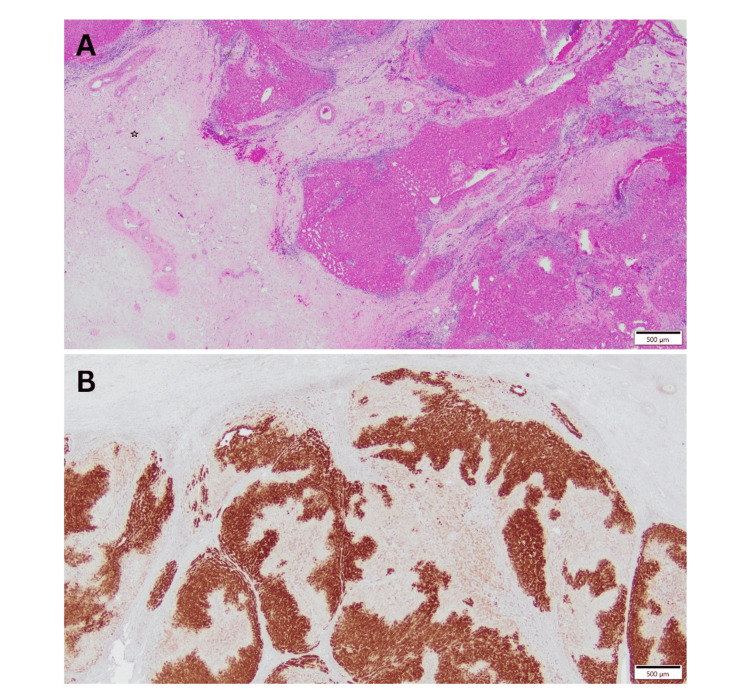
Histologic images: (A) H&E stain showing fibrous septa radiating from a central stellate collagenous scar (*) with associated thick-walled vessels, separating hepatocellular nodules. (B) Glutamine synthetase immunostain highlighting the characteristic map-like staining pattern within lesional nodules.

The patient recovered well from her surgery and was discharged on postoperative day 2. She had fully recovered and resumed regular activity by the time she was seen for her scheduled postoperative visit six weeks after surgery. Her follow-up MRI six months after surgical resection showed a stable right-lobe hemangioma and operative changes after a left hepatectomy.

## Discussion

Prolonged biliary stasis and abnormal flow of bile from the liver to the duodenum may result in complications such as hepatolithiasis. Clinical symptoms vary depending on the location and severity of the obstruction, but often include jaundice, pruritus, dark urine, pale stools, and right upper quadrant discomfort [1]. In some cases, particularly when obstruction develops slowly, symptoms may be absent or mild, and abnormalities may be detected incidentally during imaging performed for unrelated reasons [1].

While the majority of biliary obstructions are caused by intrinsic pathologies such as choledocholithiasis, benign or malignant strictures, or cholangiocarcinoma, extrinsic compression from adjacent masses, lymphadenopathy, or pseudocysts has also been described [4,6]. Benign hepatic lesions are rarely implicated in biliary obstruction, and to our knowledge, no prior reports have described segmental intrahepatic biliary obstruction complicated by secondary stone formation as a presentation of FNH. This case, therefore, highlights a previously unreported but important clinical scenario.

FNH accounts for roughly 8% of primary hepatic tumors and is the second most common benign liver lesion after hemangiomas [8-9]. It predominantly affects women, with peak incidence between the third and fifth decades of life [8]. Our patient fits this demographic profile by age and sex. Additionally, she had a remote history of taking oral contraceptive pills for 10 years and had started estrogen supplementation two months before presentation to our clinic due to perimenopausal symptoms, factors often implicated, though not conclusively, in FNH development. Although estrogen exposure has been proposed as a potential factor influencing FNH growth or symptoms, its etiologic role remains uncertain, as FNH has also been reported in men, children, and women who had no exposure to hormonal contraceptives [11]. Our patient was also found to have a 4.1 cm hemangioma in the right hepatic lobe. Although hepatic hemangiomas are not established risk factors for FNH, their coexistence may point to an underlying predisposition for benign vascular lesions, consistent with the hypothesis that FNH represents a hyperplastic response to localized vascular malformations [13]. Still, the precise pathogenesis of FNH remains incompletely understood.

Most FNH lesions are asymptomatic and discovered incidentally during imaging performed for unrelated conditions, as was the case in our patient, who initially underwent a CT scan for cardiac risk assessment. When symptoms do occur, they typically reflect mass effect on adjacent structures. However, clinically significant biliary compression is extremely rare. In our case, the FNH lesion was centered in the left lobe, abutting the left intrahepatic bile duct. The hilar location is particularly relevant; left-lobe lesions near the hepatic hilum can compress a relatively short, fixed ductal segment with limited capacity to accommodate extrinsic mass effect, such that even a modest-sized lesion can produce significant segmental biliary stasis. Although liver function tests remained normal, including ALP and GGT, imaging revealed segmental biliary ductal dilatation, raising suspicion for malignancy. Intraoperative ultrasound confirmed an ill-defined mass and unexpected intrahepatic gallstones, which were more extensive than appreciated on preoperative imaging. This suggests a chronic, insidious process of ductal compression and bile stasis, ultimately resulting in hepatolithiasis, a mechanism analogous to that seen in benign biliary strictures and not usually associated with FNH.

A review of the literature reveals several reported cases of symptomatic FNH, with complications including hemorrhage, spontaneous rupture, torsion, and gastric outlet obstruction [12,14-18]. These cases typically involved large or exophytic lesions exerting mass effect on surrounding structures, often requiring surgical resection. The only prior report of biliary involvement was described by Bente et al. [12], where FNH caused extrahepatic compression of the cystic duct without intrahepatic ductal obstruction or stone formation. In contrast, our case demonstrates segmental intrahepatic biliary obstruction with resultant hepatolithiasis, a complication not previously described in the setting of FNH, expanding the known clinical spectrum of this typically indolent lesion.

Imaging plays a central role in the diagnosis of FNH. On contrast-enhanced CT or MRI, classic FNH features include a well-demarcated, homogeneously hyperenhancing lesion on the arterial phase with a hypointense central stellate scar that enhances on delayed imaging, and an isointense or a hyperintense signal on hepatobiliary-phase imaging when a hepatocyte-specific contrast agent is used [19]. In our patient, Clariscan (gadoterate meglumine), a standard extracellular contrast agent, was used. Hepatobiliary-phase imaging with a hepatocyte-specific agent such as gadoxetate disodium (Eovist) was not performed, which may have provided additional characterization, as FNH typically demonstrates an isointense or hyperintense signal on the hepatobiliary phase, a feature that can help distinguish it from malignant lesions. The lesion did not exhibit classic arterial hyperenhancement; instead, it demonstrated peripheral rim enhancement on the arterial phase with progressive fill-in on delayed images, a pattern more consistent with intrahepatic cholangiocarcinoma. The associated segmental biliary ductal dilation further raised suspicion for a malignant process, as biliary obstruction is not a recognized feature of FNH.

The failure of both CT and MRI to identify the intrahepatic stones preoperatively also warrants mention. Pigment stones, which are more common in the setting of chronic bile stasis, may be isodense to bile on CT and may not produce the signal void typically associated with calculi on T2-weighted MRI. Additionally, the dominant mass lesion likely drew attention away from subtle biliary findings. MRCP sequences were performed as part of the standard liver MRI protocol and demonstrated biliary ductal dilation without an identifiable obstructing lesion or calculus, further contributing to the diagnostic uncertainty and highlighting the limitations of cross-sectional imaging in detecting pigment stones within mildly dilated ducts. This diagnostic uncertainty, combined with the risk of missing a resectable malignancy, led to the decision to proceed with an anatomic left hepatic lobectomy and cholecystectomy. Histopathology ultimately confirmed a 3.5 cm FNH with no evidence of malignancy or dysplasia. Associated findings included nodular regenerative hyperplasia, intrahepatic gallstones, and chronic biliary inflammation, findings consistent with long-standing bile duct obstruction.

Management of FNH depends on symptomatology, lesion size, and diagnostic clarity. For asymptomatic patients with classic imaging features, observation is generally recommended [20]. Surgical resection is reserved for symptomatic lesions, diagnostic uncertainty, rapid growth, or complications such as hemorrhage or mass effect. In this case, the combination of segmental biliary obstruction, significant stone burden, and radiologic suspicion for cholangiocarcinoma justified surgical intervention. Awareness of this rare presentation may help prevent misdiagnosis and guide appropriate surgical management when benign hepatic lesions mimic cholangiocarcinoma due to secondary biliary complications.

## Conclusions

Although focal nodular hyperplasia is typically indolent, this case demonstrates that small lesions located near the hepatic hilum may cause clinically significant biliary obstruction and secondary hepatolithiasis. This previously unreported presentation highlights the importance of considering benign etiologies in the differential diagnosis of segmental biliary dilation with mass lesions. Multidisciplinary evaluation remains essential to guide appropriate management when benign hepatic tumors mimic malignancy.
